# Decoding colorectal cancer lung metastasis: a global research odyssey

**DOI:** 10.3389/fonc.2025.1587422

**Published:** 2025-07-24

**Authors:** Xu Zhang, Liping Wang, Tong Fu, Xuchu Duan, Hao Wang, Yuqing Chen, Xiaoyu Liu

**Affiliations:** ^1^ Key Laboratory of Neuroregeneration of Jiangsu and Ministry of Education, Co-innovation Center of Neuroregeneration, School of Life Science, Nantong Laboratory of Development and Diseases, Medical College, Department of Pharmacy, Affiliated Hospital of Nantong University, Affiliated Wuxi Clinical College of Nantong University (Wuxi No.2 People’s Hospital), Nantong University, Nantong, China; ^2^ Clinical Medical Research Center, Wuxi No.2 People’s Hospital, Jiangnan University Medical Center, Jiangnan University, Wuxi, China; ^3^ Department of General Surgery, Zhangjiagang Sixth People’s Hospital, Suzhou, China; ^4^ Department of Electrocardiography, Affiliated Hospital of Jiangnan University, Wuxi, China; ^5^ Department of Hepatobiliary and Pancreatic Surgery Affiliated Wuxi No.2 People's Hospital of Nanjing Medical University, Wuxi, China

**Keywords:** bibliometric analysis, colorectal cancer, pulmonary metastases, molecular mechanisms, surgical treatment

## Abstract

**Background:**

Colorectal cancer (CRC) remains a major global health burden, particularly when complicated by pulmonary metastasis (PM), which significantly worsens prognosis and reduces survival despite active treatment. The absence of standardized therapeutic strategies for CRC with PM has driven research into its underlying biological mechanisms, including epithelial-mesenchymal transition, immune evasion, and tumor microenvironment dynamics.

**Methods:**

A bibliometric analysis was conducted on 2,645 publications related to CRC with PM retrieved from the Web of Science Core Collection (1991–2025). Analytical tools such as CiteSpace and VOSviewer were employed to map the intellectual structure, collaboration networks, and research hotspots within this domain.

**Results:**

The analysis identified China and Japan as the most prolific countries in CRC–PM research, with Robert M. Hoffman (USA) and David L. Morris (Australia) as the most productive authors, and Joachim Pfannschmidt (Germany) as the most frequently cited. Annals of Surgery was recognized as the most influential journal. Four primary research themes were identified: (1) molecular mechanisms underlying CRC lung metastasis, (2) surgical intervention strategies, (3) systemic therapies and clinical trials, and (4) clinical management and outcome evaluation. These studies reflect a growing emphasis on multidisciplinary approaches and translational research.

**Conclusion:**

The field has witnessed notable progress in multimodal treatment strategies, encompassing advances in surgical techniques, chemotherapy regimens, and targeted therapies, all of which have contributed to improved patient assessment and survival outcomes. Future research should focus on optimizing integrated therapeutic approaches and refining clinical management protocols to further improve outcomes for patients with CRC and pulmonary metastases.

## 1 Introduction

Colorectal cancer (CRC) ranks the most prevalent malignancies worldwide, being the third most commonly diagnosed cancer and the second leading cause of cancer-related mortality. It constitutes approximately 9.6% of all newly diagnosed malignancies and cancer-related deaths annually (1). Significant advancements in diagnostic techniques and therapeutic strategies have markedly improved the clinical prognosis for CRC patients (2). Radical resection, neoadjuvant therapy, and systemic chemotherapy remain the cornerstone treatment modalities, aiming to prolong survival and enhance quality of life (3–6). However, pulmonary metastasis (PM) from CRC is associated with notably poor clinical outcomes, significantly diminishing survival rates and quality of life (7, 8). As a frequent site of distant metastasis, CRC lung metastasis (CRCLM) poses substantial challenges in disease management and treatment optimization.

The lungs represent a frequent site for CRC metastasis, with approximately 10–29% of patients presenting with lung metastases at initial diagnosis or developing them during disease progression (9–12). Notably, the incidence of PM in rectal cancer is approximately 1.5 times higher than that in colon cancer (13). Despite improvements in treatment modalities, including surgical resection, chemotherapy, and targeted therapy,have improved patient survival, the prognosis for individuals with lung metastases remains poor (14). The presence of lung metastases generally indicates tumor progression to an advanced stage, complicating treatment strategies.

Research into CRC lung metastasis progresses, the critical role of molecular mechanisms, treatment strategies, and prognostic factors in patient management has become increasingly recognized (15–19). Employing bibliometric and visual analysis techniques provides valuable insights into emerging research trends, aiding in the identification of key research directions and future developments. This approach not only offers a comprehensive overview of the research landscape but also establishes a theoretical foundation for future clinical interventions.

Bibliometric analysis allows for a comprehensive and quantitative assessment of research trends, influential publications, collaborative networks, and thematic evolutions over time. In the context of clinical and translational cancer research, such analysis helps identify knowledge gaps, emerging hot spots, and leading contributors, thereby guiding future research directions and policy decisions. While systematic reviews and meta-analyses are powerful tools for synthesizing evidence on specific clinical questions, bibliometric studies offer a broader perspective on the development of an entire field or topic. They are particularly useful in exploring research landscapes, mapping scientific output, and uncovering the structure and dynamics of knowledge domains. This study aims to conduct a comprehensive bibliometric analysis of research on CRC lung metastasis from 1991 to 2025, reviewing current research trends, molecular mechanisms, treatment strategies, and prognostic factors, while forecasting emerging research hotspots. By doing so, we seek to offer novel insights into the clinical management of CRC lung metastasis and provide a reference for future scientific inquiries.

## 2 Materials and methods

### 2.1 Database and search strategy

**Figure 1:**
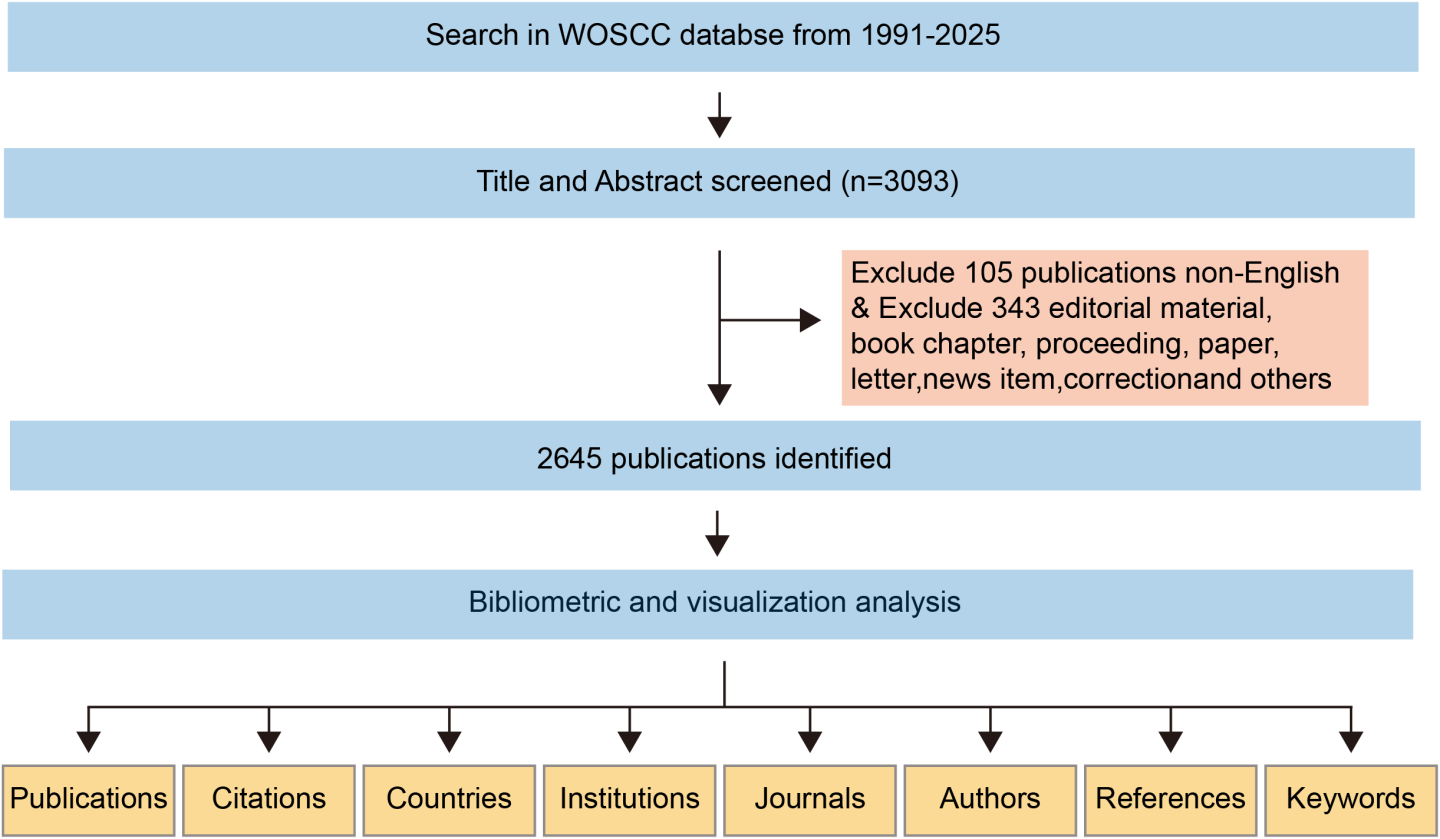
A comprehensive flowchart illustrating the stages of the publication screening process and the search methodology.

The dataset for this bibliometric analysis was obtained from the Science Citation Index Expanded (SCI-E) within the Web of Science Core Collection (WoSCC). A systematic search strategy was employed, using precisely formulated search terms applied to the “Topic” field, which includes titles, abstracts, and author-designated keywords. To ensure comprehensive data collection, no restrictions were placed on language or document type. The search terms used in the WoSCC database are as follows: TS=(((“Colorectal cancer” OR “Colorectal carcinoma” OR “Colorectal malignancy” OR “Cancer of the colon and rectum” OR “Intestinal cancer” OR “Colon cancer” OR “Rectal cancer” OR “Rectosigmoid cancer”) AND (“Pulmonary metastasis” OR “Pulmonary metastases” OR “Lung metastasis” OR “Lung metastases” OR “Metastatic disease in the lungs” OR “Secondary lung tumors” OR “Metastatic lesions in the lungs”)) OR (“Colorectal cancer with pulmonary metastasis” OR “Colorectal cancer with pulmonary metastases” OR “Pulmonary metastases secondary to colorectal cancer” OR “Lung metastasis originating from colorectal cancer” OR “Metastatic colorectal cancer to the lungs” OR “Pulmonary metastatic colorectal carcinoma” OR “Isolated lung metastasis from colorectal carcinoma”)). A total of 3,093 publications were identified through an exhaustive database search. After excluding 105 non-English publications and 343 non-research materials (e.g., book chapters, conference proceedings, letters, news articles, and errata), the final dataset consisted of 2,645 original English-language research articles and review papers related to colorectal cancer lung metastasis. To ensure the reliability and completeness of the dataset, the search was finalized on January 23, 2025, minimizing the risk of data loss due to subsequent database updates. The search strategy and screening process are illustrated in **Figure 1**. Only peer-reviewed, original English-language research articles and review papers were considered eligible for inclusion in this study.

### 2.2 Data analysis

Data on titles, keywords, authors, abstracts, publication years, citations, references, countries/regions, and institutions were carefully extracted from the WoSCC database and stored in plain text format for analysis. The 2021 edition of the Journal Citation Reports (JCR) was used to determine journal impact factors with high precision. For visualization, the Java-based bibliometric tool VOSviewer, recognized for its advanced analytical capabilities, was employed. Developed by Van Eck and Waltman in 2009 at Leiden University’s Centre for Science and Technology Studies (CWTS), VOSviewer is acclaimed for its exceptional graphical functionalities. This tool is particularly well-suited for sophisticated bibliometric analyses (20, 21). Detailed instructions for using VOSviewer are available at https://www.vosviewer.com/getting-started. CiteSpace, an advanced bibliometric tool developed by Professor Chaomei Chen at Drexel University, USA, was also utilized to perform detailed analyses of productive authors, keywords, institutions, countries/regions, co-cited references, and journals. CiteSpace enabled the identification of citation bursts, timeline trends, reference citation dynamics, and dual-map overlays, facilitating the visualization of emerging keyword patterns and trends (22, 23). It played a critical role in mapping foundational knowledge, uncovering research hotspots, and forecasting emerging research frontiers related to pulmonary metastases from colorectal cancer (24). Comprehensive tutorials for using CiteSpace are available at https://citespace.podia.com/. Statistical analyses and the creation of line charts to illustrate annual publication trends were carried out using Microsoft Excel, ensuring both accuracy and clarity.

## 3 Results

### 3.1 Quantitative analysis of publications and citations

**Figure 2:**
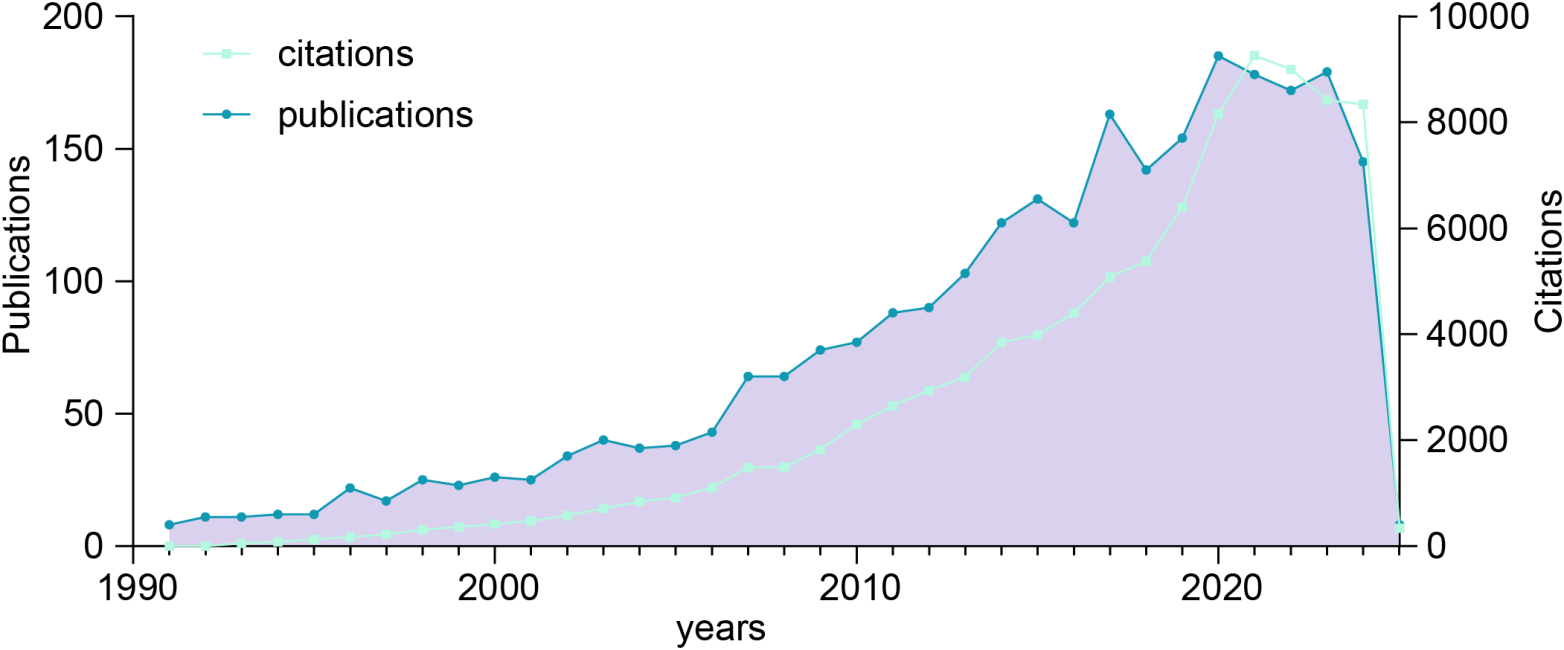
Trends in the annual publication and citation counts for colorectal cancer with pulmonary metastases from 1991 to 2025.

The volume of publications over a defined period serves as a key indicator of research progress and helps identify developmental trends within a specific field (25). As shown in **Figure 2**, an exhaustive screening process identified 2,645 publications on colorectal cancer pulmonary metastases published between 1991 and 2025, providing valuable insights into trends in publication volume and citation patterns. The analysis revealed a steady increase in publication output over this period, reflecting growing research interest and activity in this domain. Initially, publication numbers were low in 1991, indicating the early stage of research, this period marked the beginning of significant academic interest in the subject. After 2001, publication output saw substantial growth, with notable peaks in 2003, 2007, 2015, 2017, 2020, and 2023. These peaks likely correspond to key technological advancements or seminal research milestones that spurred further investigations. Notably, 2023 experienced an unprecedented surge in publications, signaling a heightened academic focus and accelerated progress in this research area. Citation trends mirrored this upward trajectory, reflecting the increasing recognition and influence of the field. Citation counts peaked around 2021, suggesting that earlier research garnered considerable academic attention and contributed to practical applications during this period.

### 3.2 Contributions analysis: prolific authors and co-cited scholars

As shown in **Table 1**, publication count and citation impact are key indicators of a researcher’s scholarly influence. Glenn, Derek has amassed 1,082 citations from 14 papers, averaging 77.29 citations per article—far exceeding peers. Similarly, Treasure, Tom and Morris, David, with averages of 54.43 and 43.7 citations, respectively, demonstrate significant academic recognition. The average publication year reflects research relevance; recent contributors like Kee, Ji-Ye (2019) and Mun, Jeong-Geon (2019) are emerging leaders, while Glenn, Derek’s 2009 research maintains lasting influence. Among the top 10 authors in this field, four are based in South Korea, highlighting its growing academic presence. Additionally, co-citation analysis reveals strong contributions from France and Germany, underscoring the importance of international collaboration.

**Table 1:** The 10 most prolific and frequently co-cited authors in colorectal cancer metastasis research.

rank	Name	h-index	Documents	Citations	Average Publication Year	Average citations
1	Hoffman, Robert m.	12	19	663	2014	34.8947
2	Morris, David	12	19	831	2011	43.7368
3	Kee, Ji-Ye	12	16	404	2019	25.25
4	Kawai, Kazushige	7	15	180	2016	12
5	Nozawa, Hiroaki	7	15	134	2018	8.9333
6	Treasure, Tom	12	14	762	2015	54.4286
7	Mun, Jeong-Geon	11	14	318	2019	22.7143
8	Lee, Woo Yong	9	14	428	2013	30.5714
9	Glenn, Derek	10	14	1082	2009	77.2857
10	Hong, Seung-Heon	12	13	388	2019	29.8462

**Figure 3:**
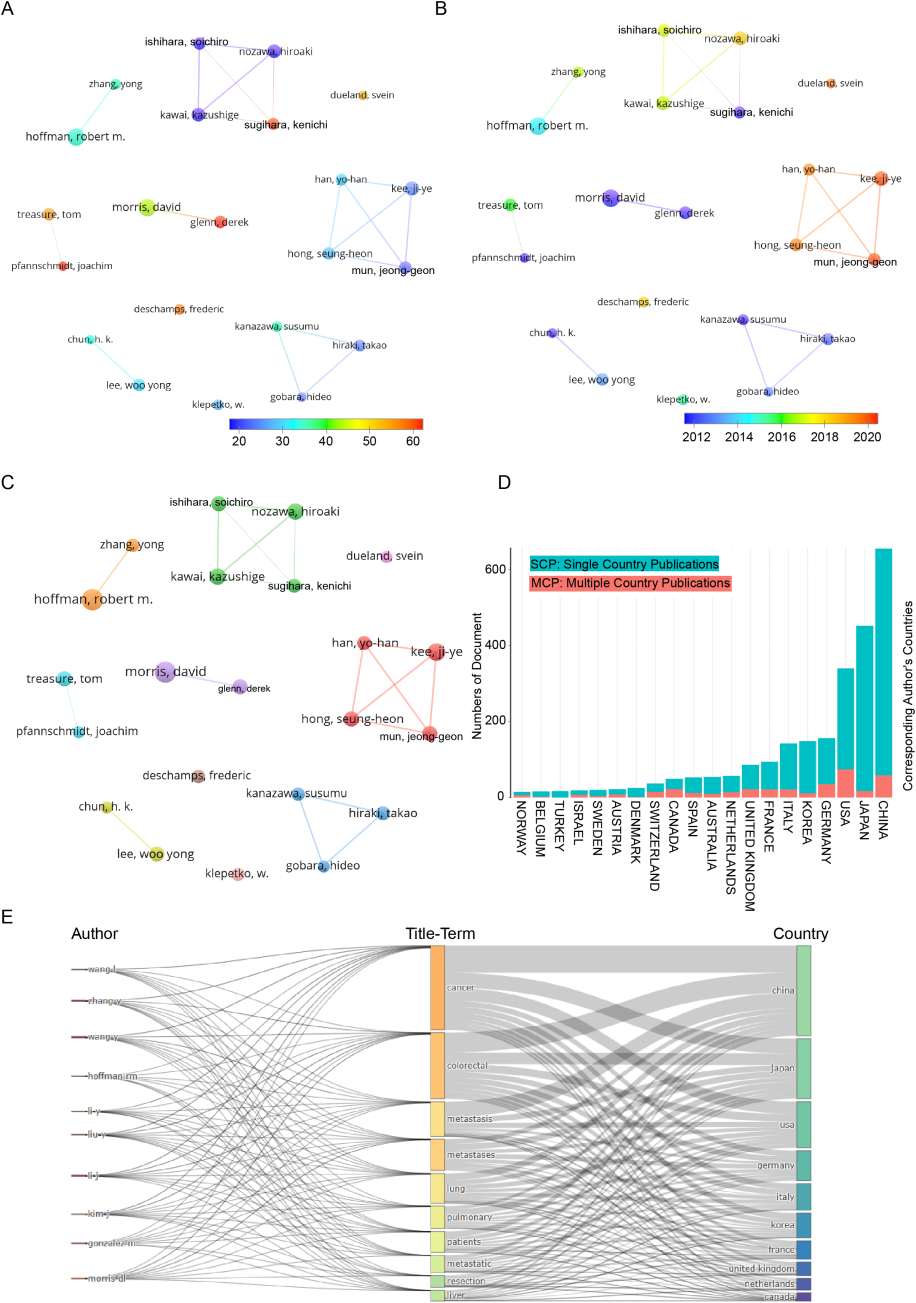
Co-authorship analysis of leading authors in the field of colorectal cancer pulmonary metastases. **(A)** Overlay visualization highlighting average citation counts. **(B)** Overlay visualization illustrating temporal trends. **(C)** Network visualization of collaborative relationships. **(D)** Dynamic patterns of international scientific collaboration. **(E)** Geographical distribution and title-term relationships of prominent authors.

**Figure 3** visualizes author collaboration networks using VOSviewer. Applying the Linlog/modularity method with a minimum threshold of 11 papers per author, we identified 17,079 authors, 22 of whom qualified for further analysis. Co-authorship clustering revealed 10 distinct groups, with node size representing publication count and edge thickness indicating collaboration strength. **Figure 3A** highlights citation impact, with a blue-to-red gradient showing influence; Sugihara, Kenichi and Han, Yo-Han are among the most cited. **Figure 3B** maps author collaborations over time, showing a shift from foundational studies (Morris, Kanazawa, Hoffman) to innovative research (Han, Hong, Mun, Kee). **Figure 3C** depicts collaboration structures, with distinct clusters: the green group (Nozawa, Ishihara, Kawai, Sugihara) as a cohesive team, the red group (Han, Hong, Mun, Kee) as highly synergistic, the blue group (Kanazawa, Gobera, Hiraki) as independent, and the orange group (Hoffman) as an influential solo network. **Figure 3D** compares domestic (SCP) vs. international (MCP) research. China leads in SCP, reflecting its domestic focus, while the U.S. dominates MCP, emphasizing global partnerships. **Figure 3E** integrates author affiliations, research topics, and geographic distribution, providing a comprehensive view of global academic collaboration.

### 3.3 Analysis of the contributions of journals

**Figure 4:**
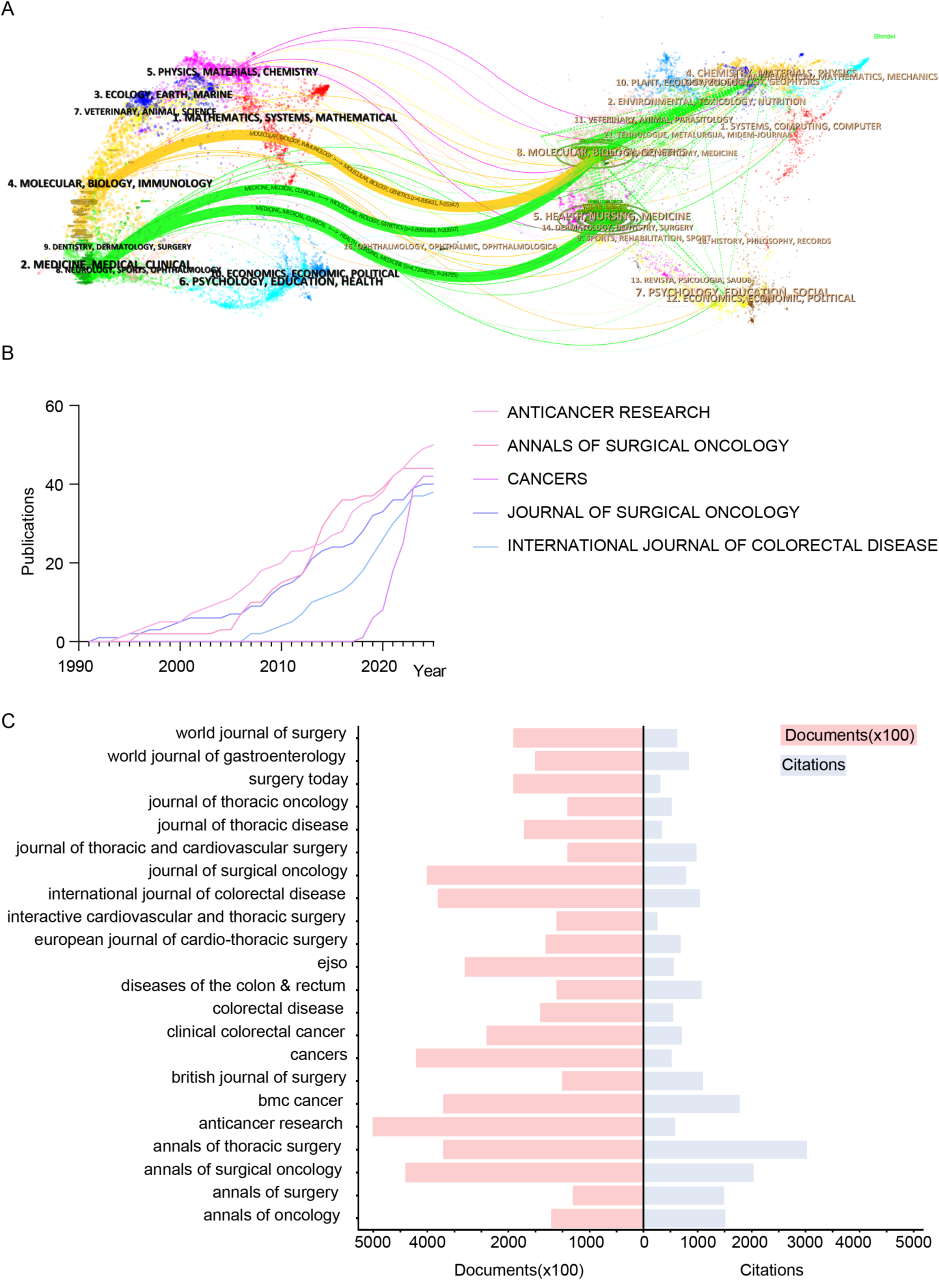
Visualization of key academic journals contributing to colorectal cancer lung metastasis research in terms of scope, influence, and publication trends. **(A)** Overlay dual-map highlighting journals related to colorectal cancer lung metastases. **(B)** Line chart analysis of the top 5 journals in this field. **(C)** Citation counts and total publication volumes of the top 22 journals.

**Figure 4A** visualizes academic journal contributions across disciplines using an overlay dual-map. The left map shows citing journals, while the right displays cited journals. Labels indicate academic fields, with colored curves representing citation trajectories. Citing journals span diverse fields, including Physics, Chemistry, Biology, Medicine, Neuroscience, Public Health, Economics, and Education. Cited journals similarly cover broad disciplines. The analysis reveals three main citation pathways: two green paths link Health, Nursing, and Medicine journals to Clinical Research, while an orange path connects Molecular Biology and Genetics to Immunology, highlighting interdisciplinary research dissemination. **Figure 4B** tracks publication trends of the top five journals in colorectal cancer lung metastasis research (1991–2025): Anticancer Research, Annals of Surgical Oncology, Cancers, Journal of Surgical Oncology, and International Journal of Colorectal Disease. Anticancer Research shows the fastest growth and is projected to lead in output by 2025. Annals of Surgical Oncology and Journal of Surgical Oncology demonstrate steady contributions, while Cancers has surged since 2017. In contrast, International Journal of Colorectal Disease has grown more slowly since 2006 due to its specialized focus. **Figure 4C** compares publication volumes and citation counts for the top 22 journals. The red bar chart represents publication volume, while the blue shows citation impact. Annals of Oncology leads in citations, reflecting its academic influence. Anticancer Research and Annals of Surgical Oncology have the highest publication volumes, driving research dissemination. Despite lower output, Annals of Surgery maintains a high citation count, underscoring its scholarly impact.

### 3.4 Analysis of the contributions by institutions

**Figure 5:**
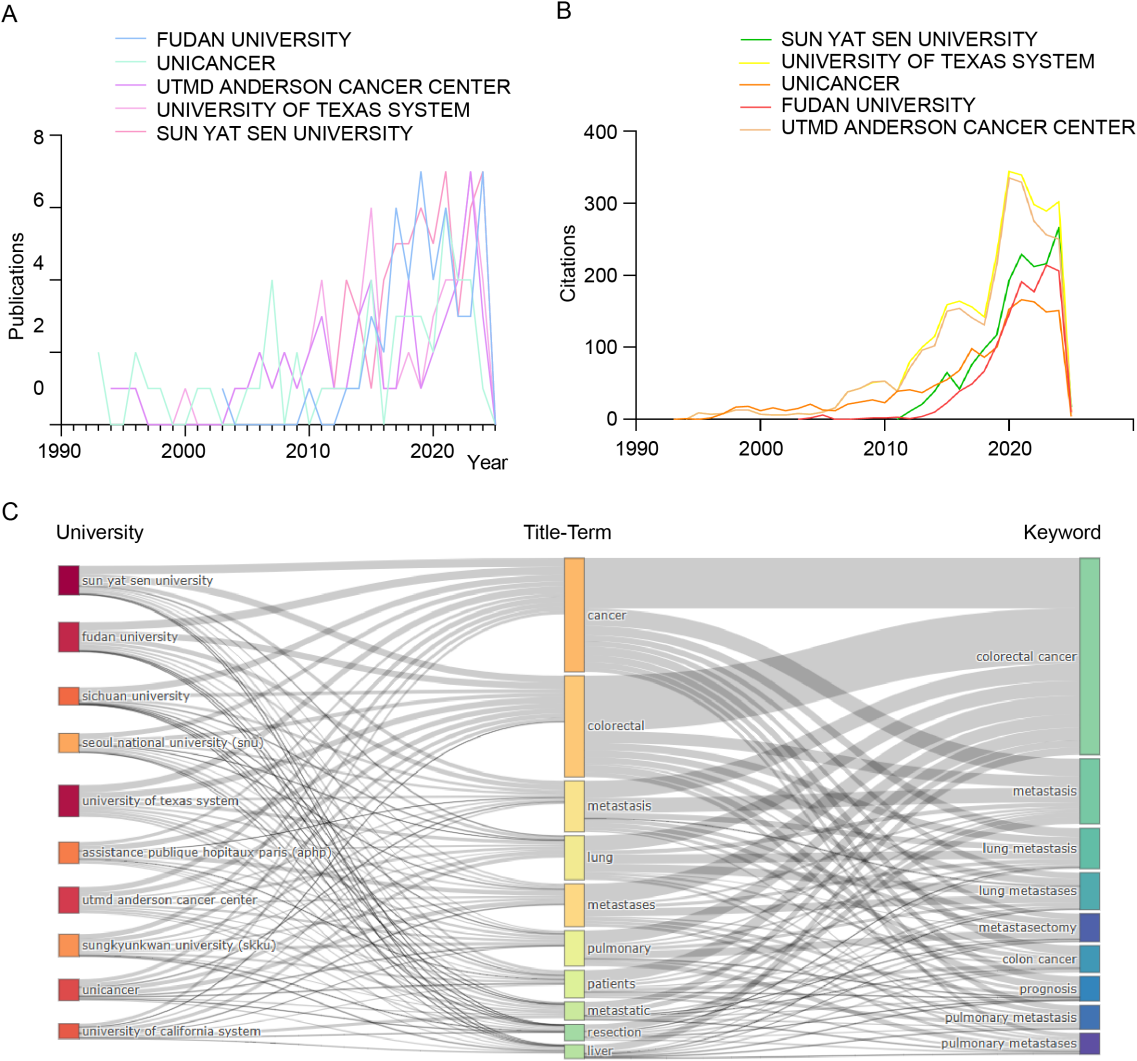
Comprehensive bibliometric analysis of key institutions contributing to the field of colorectal cancer pulmonary metastases. **(A)** Publication trends. **(B)** Citation trends. **(C)** Sankey diagram illustrating the relationships among universities, title-terms, and keywords.

**Figure 5A** analyzes publication trends of the top five institutions in colorectal cancer pulmonary metastases research (1991–2025). Fudan University and Sun Yat-sen University saw rapid growth post-2010, reflecting China’s rising contributions. In contrast, MD Anderson Cancer Center and UNICANCER made foundational contributions, while The University of Texas System’s output surged after 2014. **Figure 5B** examines citation trends, showing increased influence of Sun Yat-sen University, The University of Texas System, UNICANCER, Fudan University, and MD Anderson Cancer Center, peaking around 2020. The data highlights the continued dominance of U.S. institutions alongside China’s rapid ascent, reflecting shifting global research dynamics. **Figure 5C** presents a Sankey diagram linking institutions (left), title terms (middle), and keywords (right). Fudan and Sun Yat-sen focus on fundamental research (“cancer,” “colorectal cancer,” “metastasis”), while The University of Texas System and MD Anderson emphasize clinical applications (“pulmonary metastases,” “resection”). The diagram underscores international collaboration and the multidisciplinary nature of advancements in this field.

### 3.5 Analysis of the contributions of countries

**Figure 6:**
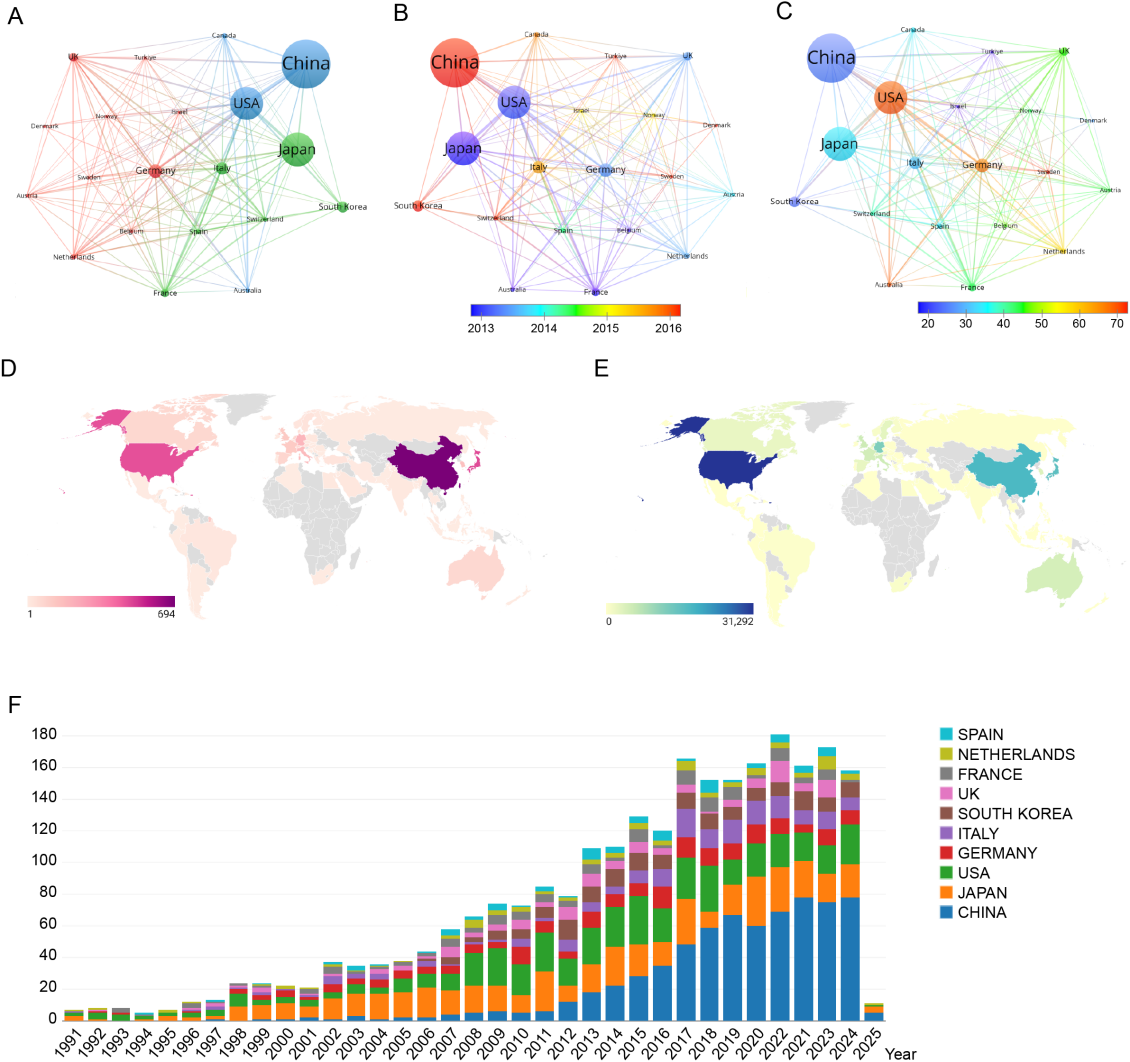
Comprehensive visualization of global research productivity, collaboration patterns, temporal trends, and citation impact in colorectal cancer lung metastasis across countries and regions. **(A)** Network visualization map showing the collaborative relationships among the top 18 most research-active countries/regions. **(B)** Overlay visualization map illustrating the average publication year across countries/regions. **(C)** Overlay visualization map highlighting the average publication output per country/region. **(D)** Geographical distribution of global citations related to colorectal cancer lung metastases. **(E)** Geographical distribution of global publications in colorectal cancer lung metastases. **(F)** Bar chart depicting the top 10 most productive countries/regions in the domain.

The VOSviewer configuration employed the Linlog/modularity method, establishing a minimum document threshold of 19 per country. Out of 70 countries and regions analyzed, 20 met this threshold. VOSviewer facilitated the grouping of these countries and regions into distinct research clusters through co-authorship analysis. In the visualization, nodes of varying colors denote separate clusters, with node sizes corresponding to each country’s publication volume, and the thickness of the connecting lines indicating the strength of academic collaboration. **Figure 6A** illustrates that from 1991 to 2025, China led the global publication output in colorectal cancer lung metastasis research. **Figure 6B** shows a significant surge in China’s research activity in recent years, reflecting a continued upward trend. **Figure 6C** reveals that the United States has the highest average citation count in this field, highlighting the considerable academic impact and significance of its research contributions. **Figures 6D, E** display the geographical distribution of 70 countries and regions engaged in colorectal cancer lung metastasis research, emphasizing their global contributions in both publication output and citation impact. Finally, **Figure 6F** outlines the publication trends of the 10 most research-active countries globally, providing key insights into the evolution of this research domain.

### 3.6 Analysis of a highly cited study

**Figure 7:**
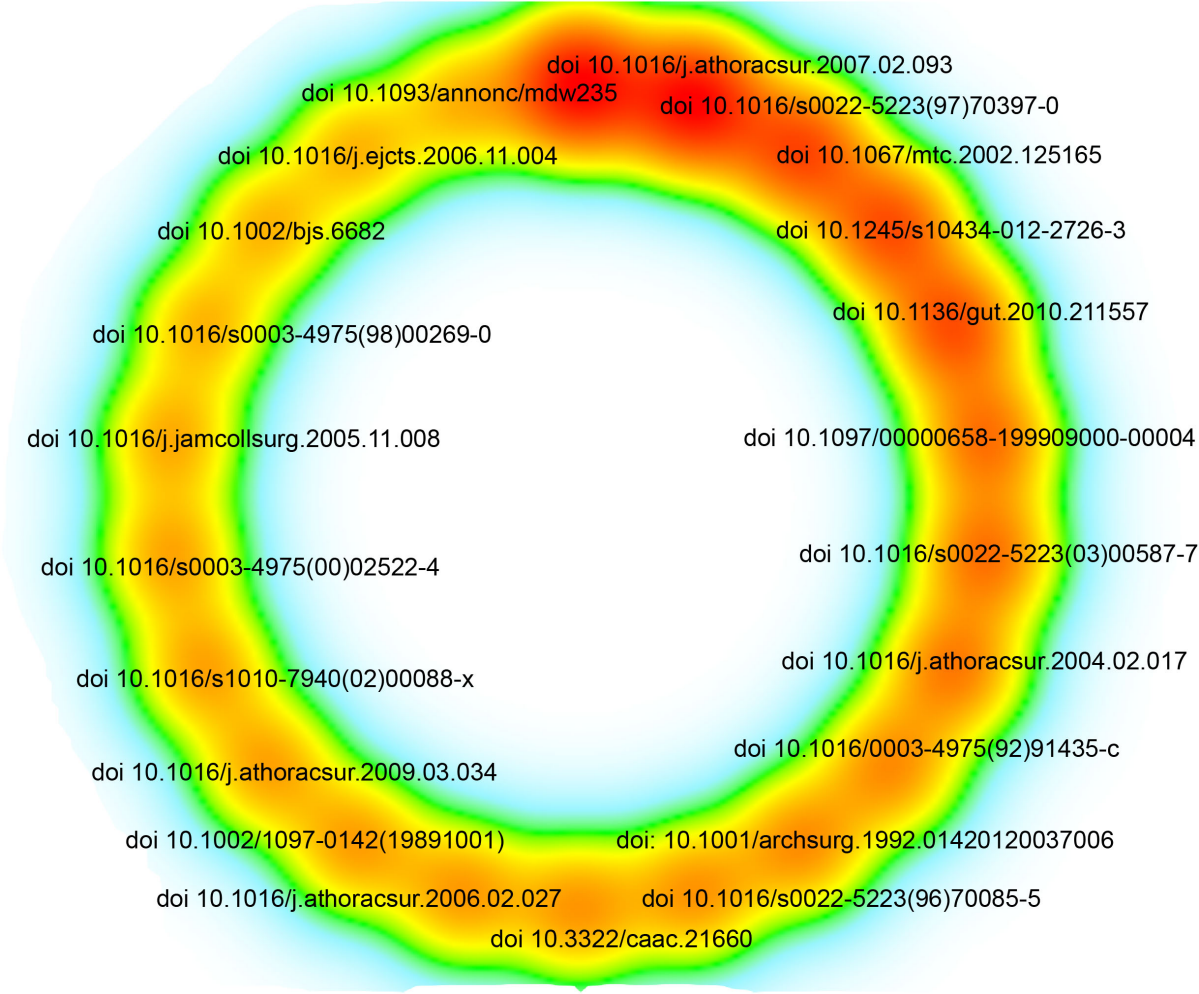
Density-based visualization of the top 22 most frequently co-cited references.

The most cited article, published in 2004 in *The Annals of Thoracic Surgery*, is titled “Surgical resection of pulmonary metastases from colorectal cancer: a systematic review of published series.”(**Figure 7**, **Supplementary table 1**) This article holds the highest citation count among all the analyzed literature, highlighting its significant impact on the field. Among the 62,907 references, 22 articles have been cited more than 87 times, as illustrated in **Figure 7** and **Supplementary table 1**.

### 3.7 Analysis of keywords

A bibliometric analysis using VOSviewer explored the research landscape of colorectal cancer lung metastases. The Linlog/modularity method was applied with a minimum occurrence threshold of 14, identifying 30 high-frequency keywords. A keyword co-occurrence network (**Supplementary Figure 1**) illustrates relationships, where link thickness represents co-occurrence frequency. The citation counts of the key research directions in **Supplementary Figure 1A** from high to low are shown current research trends in colorectal cancer lung metastasis focus on leucovorin-based chemotherapy regimens, while investigating the molecular mechanisms of invasion, growth, and pathway activation; insights from breast cancer studies contribute to understanding lung metastases, with particular emphasis on apoptosis regulation and migration dynamics in colon cancer. The use of oxaliplatin and fluorouracil remains central, as studies increasingly explore the clinical implications of pulmonary metastases, their relation to liver metastases, and the therapeutic value of metastasectomy, especially pulmonary metastasectomy, in improving patient outcomes.

**Figure 8:**
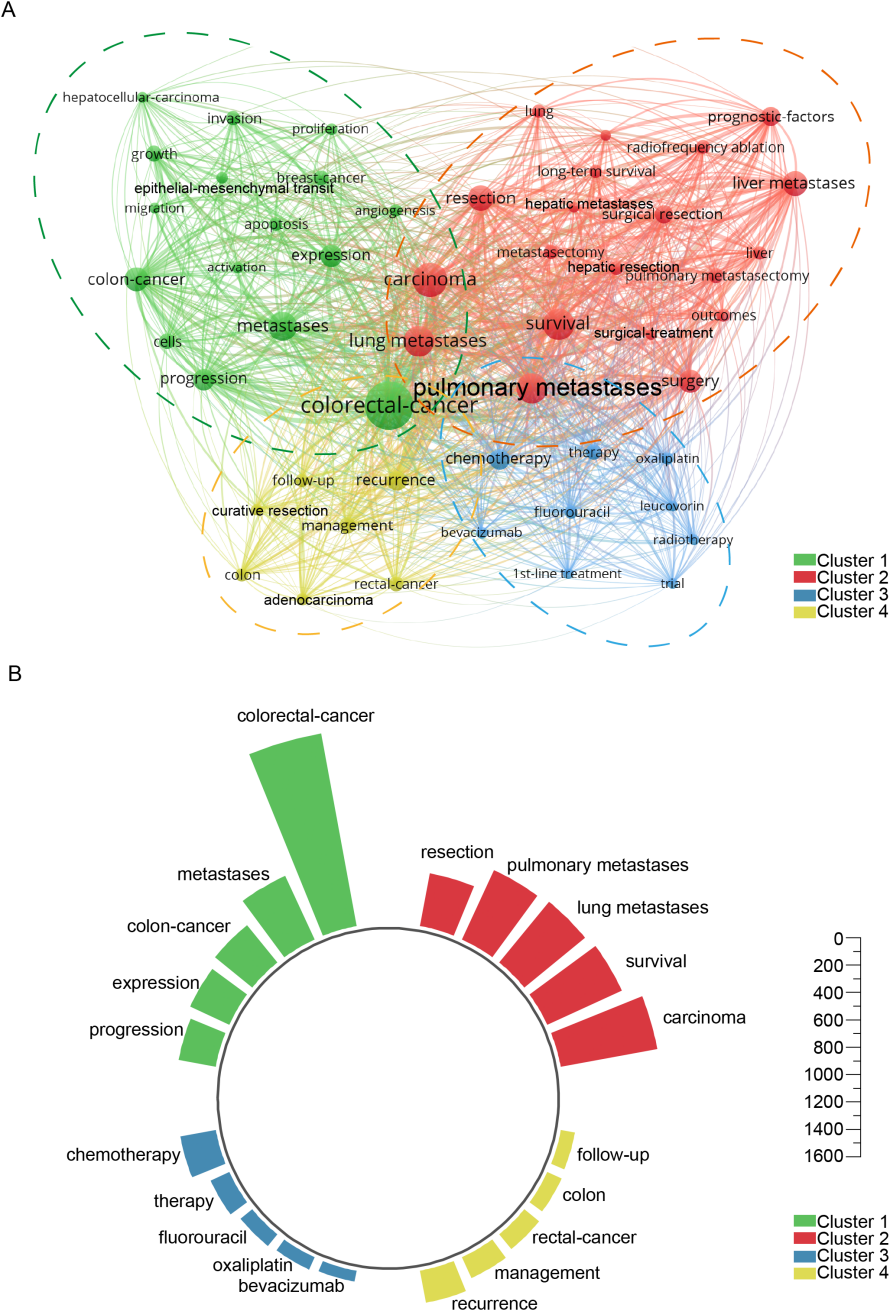
Keyword co-occurrence analysis revealing thematic clusters. **(A)** Comprehensive network visualization map. **(B)** The five most prominent and influential keywords across the four clusters.

The results of the research on hot words in **Supplementary Figure 1B** from recent years to distant years show recent studies on colorectal cancer lung metastasis increasingly emphasize tumor proliferation and patient outcomes, with growing interest in lung metastasectomy and pulmonary metastasectomy as part of multidisciplinary strategies. The incorporation of targeted agents such as bevacizumab has shown potential in improving survival, while the molecular basis of invasion continues to be explored. Surgical approaches including metastasectomy, hepatic resection, and management of pulmonary metastases and liver metastases remain central to treatment paradigms, with surgical treatment combined with chemotherapeutics like fluorouracil representing a long-standing standard of care. **Supplementary Figure 1C** highlights the top 20 keywords with the highest citation burst rates, showcasing evolving research priorities. The timeline visualization tracks keyword progression, while **Supplementary Figure 1D** maps 16 major research clusters, including lung metastases, colorectal carcinoma, MRI, and chemotherapy. Established hotspots like “Krukenberg tumor” and “immunohistochemistry” reflect sustained interest, whereas “neoadjuvant therapy” has recently gained prominence, signaling a shift toward advanced treatment strategies. **Supplementary Figure 2** examines thematic trends. **Supplementary Figure 2A** identifies key topics, with recent growth in “carcinoma,” “surgical,” and “microsatellite” research. **Supplementary Figure 2B** traces developments in pulmonary metastases, highlighting increased focus on “mouse models,” “computed tomography,” “microwave ablation,” and “clinical outcomes,” reflecting advancements in diagnostics and treatment. **Figure 8A** presents a keyword network with terms occurring over 59 times, grouped into four clusters: (1) cancer progression (e.g., invasion, proliferation, epithelial-mesenchymal transition), (2) surgical management (e.g., lung metastases, resection, survival, radiofrequency ablation), (3) systemic therapies (e.g., oxaliplatin, chemotherapy, radiotherapy), and (4) clinical outcomes (e.g., recurrence, curative resection, follow-up). **Figure 8B** ranks the five most frequent keywords in each cluster, highlighting their impact on colorectal cancer research.

## 4 Discussion

### 4.1 Global research status and trends

Based on the analysis of national publication data, China, Japan, and the United States have made substantial contributions, publishing 694, 480, and 467 articles, respectively. These three countries collectively account for 55.4% of all publications in this field. In terms of journal publications and citation metrics, the United States leads globally in colorectal cancer lung metastasis research, emphasizing the significance and influence of research papers originating from U.S.-based institutions. Additionally, an analysis of total author collaboration strength reveals that the top four authors are based in South Korea, indicating the formation of a strong and influential academic community within the country. Authors ranked 5th to 10th primarily come from Japan, further suggesting that Japan has established a well-connected and influential research network. The analysis of the top five research institutions shows that two are based in China, two in the United States, and one in France. These institutions have played a pivotal role in advancing research on colorectal cancer lung metastasis, highlighting the trends of global collaboration and academic competition.

### 4.2 Research keyword analysis

Cluster analysis of 52 keywords that appeared more than 59 times categorizes them into four main clusters: Cluster 1 - green cluster (**Figure 8**), focusing on the molecular and biological mechanisms of colorectal cancer lung metastasis; Cluster 2 - red cluster (**Figure 8**), centering on the surgical treatment of colorectal cancer lung metastasis; Cluster 3 -blue cluster (**Figure 8**), covering systemic therapies and related clinical trials; and Cluster 4 - yellow cluster (**Figure 8**), addressing clinical management and treatment outcomes. The co-occurrence analysis of these keywords highlights key research directions and emerging trends in the field (**Supplementary Figure 1**). The timeline analysis of keywords and the top 20 highlighted terms reveals that from 1991 to 2025, keywords related to molecular mechanisms, surgical treatments, and prognosis have been central to the field, indicating their long-standing importance. In recent years, research on chemotherapy has emerged as a critical focus, often intersecting with prognosis analysis (**Supplementary Figure 2**).

High-frequency cited terms such as leucovorin, oxaliplatin, and fluorouracil highlight the central role of chemotherapy backbones in historical and ongoing treatment strategies. Meanwhile, frequent mentions of invasion, growth, activation, apoptosis, and migration underscore sustained interest in the molecular mechanisms driving metastasis. The inclusion of breast cancer as a comparative model reflects the translational cross-talk between cancer types. Furthermore, terms like lung metastases, pulmonary metastases, liver metastases, and metastasectomy emphasize the clinical relevance of organ-specific dissemination and surgical intervention, with pulmonary metastasectomy emerging as a key procedural focus. In contrast, a chronological trend analysis—from most recent to older publications—shows a shift toward evaluating proliferation, outcomes, and survival as metrics of therapeutic success, accompanied by increasing attention to personalized surgical approaches like lung metastasectomy and pulmonary metastasectomy. The rising use of bevacizumab indicates a growing role of targeted therapies, while hepatic resection and surgical treatment continue to be refined through integrated multimodal approaches. Although fluorouracil remains a foundational agent, current research prioritizes combination strategies and biologically informed selection.

Together, these patterns reflect a transition from single-modality interventions toward a multidisciplinary paradigm that incorporates molecular profiling, surgical innovation, and real-world outcome assessments. This evolution not only redefines research priorities, focusing on metastatic biology and organotropism, but also directly informs clinical practice by emphasizing individualized, evidence-based treatment planning for patients with metastatic colorectal cancer. This trend aligns with the findings of our cluster analysis (**Figure 8**). This paper provides an in-depth discussion of the four clusters, aiming to uncover critical areas and future research directions in the study of colorectal cancer lung metastasis.

### 4.3 Green cluster: molecular and biological mechanisms underpinning colorectal cancer lung metastasis

Colorectal cancer lung metastasis is a complex, multi-stage process characterized by critical molecular and biological mechanisms that govern its initiation and progression. These mechanisms involve not only the invasion, migration, and proliferation of cancer cells but are also intricately linked to essential processes such as alterations in the tumor microenvironment (TME) and immune evasion. Recent studies indicate that the ability of cancer cells to invade and migrate is a hallmark of tumor metastasis, with epithelial-mesenchymal transition (EMT) playing a central role in facilitating tumor cell migration to the lungs. Understanding of these molecular mechanisms is paramount for the development novel therapeutic strategies.

The TME encompasses the complex interactions between tumor cells and their surrounding environment, playing a vital role in tumor initiation, progression, and metastasis. Tumor cells secrete signaling molecules in autocrine or paracrine manners, and within the TME, intercellular interactions and soluble signals regulate EMT, promoting tumor invasion and metastasis (26, 27). EMT enables colorectal cancer cells to acquire the ability to migrate and invade, allowing them to transition from tightly adherent epithelial cells to mesenchymal-like cells capable of invading blood vessels and spreading to distant organs (28). For instance, Zhen Bao’s research on the role of SNAIL in EMT demonstrated its promotion of CXCL2 secretion by mesenchymal cells, which enhances the infiltration of M2 macrophages and drives tumor metastasis (29). Key signaling pathways such as TGF-β, Wnt/β-catenin, and Notch are recognized as major regulators of EMT, with their aberrant activation contributing to colorectal cancer metastasis and drug resistance (30–33). During EMT, the downregulation of E-cadherin and the upregulation of N-cadherin and Vimentin are strongly associated with the metastatic potential of cancer cells (34, 35).

Secondly, the invasion and migration of cancer cells are hallmark characteristics of colorectal cancer lung metastasis. Invasion requires cancer cells to degrade the extracellular matrix (ECM), a process largely facilitated by matrix metalloproteinases (MMPs). MMP-2 and MMP-9, members of the MMP family, are strongly linked to colorectal cancer metastasis. Overexpression of these proteins not only enhances tumor invasiveness but also increases the risk of metastasis (36–38). Additionally, cancer cells enhance their migratory capacity through pseudopodia formation and cytoskeletal reorganization. The activation of Rho family small GTPases plays a central role in this process, driving changes in cell morphology and enhancing their ability to migrate (39, 40).

Apoptosis inhibition is another critical factor that cancer cells must overcome during metastasis. Under normal conditions, apoptosis is a fundamental mechanism that maintains cellular homeostasis. However, in colorectal cancer cells, apoptotic pathways are often suppressed, allowing cancer cells to evade programmed cell death and sustain proliferation throughout metastasis (41). Aberrant activation of anti-apoptotic pathways, including Bcl-2 family proteins, the PI3K/AKT pathway, and NF-κB, is a key driver of this suppression (42, 43). These anti-apoptotic signals also facilitate immune evasion, allowing cancer cells to avoid host immune surveillance and continue their metastatic progression. Understanding the molecular mechanisms behind this process provides novel therapeutic targets for cancer treatment, particularly in the early stages of lung metastasis.

Gene expression and regulatory mechanisms are also pivotal in colorectal cancer metastasis. Tumor cells increase their metastatic potential by dynamically regulating the expression of genes associated with proliferation, invasion, and immune evasion (44, 45). For example, Zhongxing Liang and Tripti Khare identified that upregulation of VEGF and CXCR4 enhances tumor angiogenesis and promotes cancer cell colonization in distant organs, including the lungs, via the CXCL12/CXCR4 axis (46, 47). With the continued advancement in our understanding of these molecular mechanisms, targeted therapies designed to modulate tumor cell migration, proliferation, invasion, and apoptosis evasion present promising avenues for the development of innovative treatment strategies in colorectal cancer, particularly for patients with advanced metastatic disease.

### 4.4 Red cluster: surgical approaches in colorectal cancer lung metastasis

Surgical intervention for lung metastases represents a crucial component in managing colorectal cancer patients, necessitating a thorough prognostic evaluation being essential to developing the most effective treatment strategy. Research indicates that pulmonary metastasectomy is a primary surgical approach that enhances the long-term survival of patients with colorectal cancer lung metastases (48, 49). This procedure enables effective resection of metastatic lesions in the lungs, contributing to prolonging survival and improving the quality of life. The success of metastasectomy depends on various factors, including tumor stage, the number and size of metastatic lesions, their anatomical distribution, and the completeness of surgical margins (50, 51). Research has demonstrated that patients diagnosed at an early stage and receiving prompt surgical intervention—particularly those with a limited number of metastases and no extensive involvement of other organs—experience a more favorable prognosis (52). Clinical data suggest that patients who undergo pulmonary metastasectomy, especially those without multiple metastases or involvement of other organs, may achieve a five-year survival rate exceeding 50% (53). Additionally, multicenter studies have shown that some patients can achieve prolonged survival after surgical resection, further highlighting the therapeutic value of pulmonary metastasectomy for appropriately selected patients (54).

Radiofrequency ablation (RFA), an emerging minimally invasive therapeutic approach, has shown promising clinical efficacy for colorectal cancer patients with lung metastases who are not candidates for surgical intervention (55). RFA destroys metastatic lung lesions through localized tissue heating, alleviating symptoms and achieving disease control (56). Compared to conventional surgical resection, RFA is less invasive, requires a shorter recovery time, and is especially suitable for high-risk patients or those with significant comorbidities. However, its therapeutic efficacy is generally inferior to surgical resection, and its clinical indications require further validation. In cases involving multiple or large metastatic lesions or deeply located lung metastases, the effectiveness of RFA may be limited (57–59).

Prognostically, several factors are key determinants of treatment outcomes in colorectal cancer patients with lung metastases. Tumor staging, metastatic burden, distribution, overall patient health, and the presence of extra-pulmonary metastases prior to surgery all influence surgical success and postoperative survival (60, 61). Preoperative tumor marker profiles, radiographic findings, and the patient’s immune status are critical parameters for guiding personalized treatment strategies (62, 63). As precision oncology continues to advance, the integration of molecular biomarker profiling, surgical intervention, and adjuvant therapies is expected to enhance survival outcomes and therapeutic efficacy for patients with colorectal cancer lung metastases.

In conclusion, the surgical management of colorectal cancer lung metastases remains a cornerstone for improving patient survival. With the advent of novel therapeutic approaches, such as radiofrequency ablation, the integration of surgery with minimally invasive techniques offers expanded treatment possibilities. Further refinement of surgical strategies and improved prognostic assessment will enhance treatment outcomes and extend survival for these patients.

### 4.5 Blue cluster: systemic therapies and associated clinical trials in colorectal cancer lung metastasis

Patients diagnosed with advanced colorectal cancer present with significant distant metastases at the time of initial diagnosis or have tumors that are resistant to surgical resection. For these individuals, chemotherapy may represent the only viable option to extend survival. The combination of oxaliplatin or irinotecan with fluorouracil, often administered alongside folate-based drugs like leucovorin, has been shown to improve clinical outcomes, slow disease progression, and modestly increase patient survival (64). Bevacizumab, a targeted monoclonal antibody, inhibits tumor angiogenesis and provides an alternative therapeutic approach, particularly for patients who are resistant to chemotherapy. However, its clinical use is still in the early stages, requiring further investigation to establish its long-term therapeutic efficacy. While long-term data are lacking to confirm the efficacy of bevacizumab in combination with oxaliplatin, ongoing studies continue to explore the potential clinical benefits of these combination therapies (65, 66).

As clinical trials progress, novel therapeutic regimens and pharmacological agents are being gradually integrated into clinical practice. Numerous studies have demonstrated that combining chemotherapy with radiotherapy can enhance therapeutic effectiveness. In patients with locally advanced lung metastases, radiotherapy has shown significant benefits in tumor growth control and symptomatic relief (67–69). Although chemotherapy and targeted therapies have made considerable advances in clinical practice, treatment responses vary among individuals, resulting in inconsistent outcomes (70, 71). This variability highlights the need for future research to refine therapeutic strategies, particularly through the integration of tumor molecular profiling and patient-specific characteristics to develop more personalized treatment regimens. As systemic therapies and clinical trials continue to evolve, treatment options for colorectal cancer lung metastases are becoming increasingly diverse and complex. By considering various therapeutic modalities and tailoring treatments to individual patient needs, future advancements hold the potential to significantly improve survival outcomes for these patients.

### 4.6 Yellow cluster: clinical management strategies and treatment outcomes in colorectal cancer lung metastasis

Postoperative management and follow-up strategies play a crucial role in reducing recurrence rates and optimizing patient prognosis. Studies have shown that routine high-resolution imaging (e.g., CT, PET-CT) and monitoring tumor markers (such as CEA and CA19-9) enable early detection of recurrent lesions, facilitating timely secondary interventions or surgery (72–74). Several clinical trials have confirmed that adjuvant therapy following surgery, including chemotherapy, targeted therapy, and immunotherapy, significantly reduces the risk of recurrence and improves survival outcomes in high-risk patients. Specifically, for patients with rectal cancer lung metastases, individualized treatment regimens combining postoperative chemotherapy and targeted therapy are emerging as a mainstream strategy to further enhance therapeutic results (75, 76).

Despite these advancements, recurrence rates among patients with lung metastases remain high, with many experiencing local or distant recurrence within 1 to 3 years post-surgery (77). Research has identified critical risk factors for recurrence, including the invasiveness and biological characteristics of the primary tumor, the number and size of lung metastases, and elevated preoperative CEA levels (78, 79). Therefore, personalized follow-up strategies for high-risk patients, including more intensive imaging surveillance and dynamic biomarker monitoring, can improve early detection of recurrence and provide crucial guidance for subsequent treatment decisions. As precision medicine progresses, future postoperative management will increasingly incorporate molecular subtyping and genetic mutation analysis to refine treatment strategies, ultimately improving long-term survival and quality of life for patients with colorectal cancer lung metastases.

### 4.7 Treatment and management strategies

The management of CRC lung metastasis involves a multidisciplinary approach that integrates surgical, ablative, and systemic therapies. Among local treatments, pulmonary metastasectomy and radiofrequency ablation are the most commonly employed strategies, especially for oligometastatic disease.

Pulmonary metastasectomy has long been considered the standard of care for selected patients with resectable lung metastases and favorable performance status. Numerous retrospective studies and institutional series have demonstrated a survival benefit, with reported 5-year overall survival rates ranging from 30% to 50% in carefully selected patients (80, 81). The procedure is typically indicated for patients with: Controlled primary tumor, Limited number of metastases, No extrapulmonary disease, Adequate cardiopulmonary reserve. Despite its widespread use, robust randomized controlled trial evidence is lacking. Radiofrequency Ablation has emerged as a minimally invasive alternative for patients deemed unfit for surgery or those with unresectable disease. It delivers thermal energy to induce coagulative necrosis of tumor tissue. Several studies have shown that Radiofrequency Ablation can achieve satisfactory local control and acceptable survival outcomes in selected cases (82, 83). A meta-analysis concluded that radiofrequency ablation combined with chemotherapy significantly improves overall survival compared to chemotherapy alone in patients with pulmonary metastases (84). Retrospective comparisons suggest that both approaches can offer meaningful survival benefits in selected patients. Metastasectomy may offer superior local control, while radiofrequency ablation provides the advantage of reduced morbidity, shorter hospital stays, and repeatability. Patient selection remains critical in choosing the optimal approach. Surgery is preferred for patients with resectable lesions and good functional status, while radiofrequency ablation may be more suitable for those with high surgical risk or multifocal disease not amenable to resection. Fenton, H.M. et al. reported significant variations in the incidence of lung metastasectomy for colorectal cancer across the United Kingdom’s National Health Service (85). Treasure, T. et al., through observational studies, demonstrated that the survival rate without metastatic tumor resection is higher than commonly assumed. The observed survival differences are largely attributable to patient selection biases. They caution against inferring the impact of metastatic tumor resection on survival rates without accounting for these biases (86). The retrospective analysis by Zurlo IV et al. suggested that surgical intervention for lung metastases may improve survival outcomes (87). Ziranu, P. et al. highlighted that the Meta-Lung Score offers valuable prognostic insights specifically for lung metastases originating from colorectal cancer (88). Wang, R. et al. developed a predictive model integrating machine learning, pathological features, radiomics, immune scores, and clinical characteristics, which reliably forecasts overall survival and disease-free survival in patients with colorectal cancer pulmonary metastasis following surgery (89).

Ongoing Clinical Trials: Several ongoing trials are investigating the role of local therapies in metastatic CRC, although most focus on hepatic metastases. These include: COLLISION Trial (NCT03088150): The primary objective is to prove non-inferiority of thermal ablation compared to hepatic resection in patients, providing a model that could be translated to lung metastases. Chinese multicenter prospective studies (ChiCTR-ORC-17012904) are ongoing to evaluate clinical application of CT-guided microwave ablation in the treatment of pulmonary metastases from colorectal cancer. Such studies are expected to shed light on treatment selection and define the role of ablative strategies in the context of systemic control. Guidelines and Recommendations: According to the NCCN Guidelines, metastasectomy is recommended for patients with resectable disease and adequate functional status. RFA or stereotactic body radiotherapy (SBRT) may be considered for unresectable or high-risk surgical candidates. The Chinese Society of Clinical Oncology (CSCO) also supports local ablative treatments such as RFA for patients who are inoperable or have limited metastatic disease, especially when systemic disease is controlled.

## 5 Limitations

This study has certain limitations. First, We fully acknowledge that limiting our review to English-language publications may introduce selection bias, particularly by underrepresenting research from non-English-speaking regions. And We strictly followed the search strategy of the article and explained it in the limitation section of the article. 4% of the parts that were insufficient were excluded and included in the annotation. The research results did not affect the key research and guiding strategies of the article. Second, recent high-quality literature may not fully reflect its impact due to shorter publication timelines and lower citation frequencies. Despite these limitations, this study offers valuable insights into the research themes, emerging hotspots, and developmental trends in colorectal cancer lung metastasis.

## 6 Conclusion

Through bibliometric analysis of literature on colorectal cancer lung metastasis, this study systematically assessed relevant data from various countries, institutions, authors, publication years, research disciplines, and journals, while also investigating research themes and future directions in this field. The results indicate that research in this area began in 1991 and has progressively developed into a significant research direction. This study not only provides foundational insights into the research landscape but also highlights potential collaboration opportunities for scholars seeking to explore further. We have identified four core directions and their developmental trends in colorectal cancer lung metastasis research: (1) Molecular mechanism studies, (2) Surgical treatment and prognosis analysis, (3) Systemic therapies and related clinical trials, and (4) Clinical management strategies. Among these, molecular mechanisms and surgical treatments have emerged as focal points, while advancements in chemotherapy and prognosis analysis are gaining increasing scholarly attention. The synergistic application of these approaches contributes to the advancement of personalized patient assessment. Moreover, the expansion of surgical indications and the standardization of clinical management strategies have substantially improved patient survival. The integration of chemotherapy and targeted therapies has enabled some critically ill patients to benefit from surgical intervention. In the future, optimizing and advancing the combination of surgery, radiotherapy, chemotherapy, and comprehensive clinical management strategies will be a primary research focus in this domain.

## Data Availability

The original contributions presented in the study are included in the article/**Supplementary Material**. Further inquiries can be directed to the corresponding authors.

## Supplementary material

Supplementary Figure 1 Visualization analysis of keyword citation impact, research hotspot evolution, and temporal trends in studies on colorectal cancer lung metastases. **(A)** Overlay visualization of the average citation counts for the selected keywords. **(B)** Overlay visualization depicting the average publication year of the keywords. **(C)** Top 20 keywords exhibiting the highest citation bursts. **(D)** Timeline and keyword clustering diagram illustrating research trends in colorectal cancer lung metastases.

Supplementary Figure 2 Thematic trends and their temporal evolution in colorectal cancer lung metastasis research. **(A)** Emerging research trends in the field. **(B)** Advancements in research within the field and related domains.

Supplementary Table 1 The top 22 co-cited references related to colorectal cancer lung metastasis.

## References

[1] Bray F Laversanne M Sung H Ferlay J Siegel RL Soerjomataram I. Global cancer statistics 2022: GLOBOCAN estimates of incidence and mortality worldwide for 36 cancers in 185 countries. CA: A Cancer J Clin. (2024) 74:229–63. doi: 10.3322/caac.21834, PMID: 38572751

[2] Dekker E Tanis PJ Vleugels JLA Kasi PM Wallace MB. Colorectal cancer. Lancet. (2019) 394:1467–80. doi: 10.1016/S0140-6736(19)32319-0, PMID: 31631858

[3] Simunovic M Sexton R Rempel E Moran BJ Heald RJ. Optimal preoperative assessment and surgery for rectal cancer may greatly limit the need for radiotherapy. Br J Surg. (2003) 90:999–1003. doi: 10.1002/bjs.4210, PMID: 12905555

[4] Delaney CP Kiran RP Senagore AJ Brady K Fazio VW. Case-matched comparison of clinical and financial outcome after laparoscopic or open colorectal surgery. Ann Surg. (2003) 238:67–72. doi: 10.1097/01.sla.0000074967.53451.22, PMID: 12832967 PMC1422656

[5] Gunderson LL Haddock MG Schild SE. Rectal cancer: Preoperative versus postoperative irradiation as a component of adjuvant treatment. Semin Radiat Oncol. (2003) 13:419–32. doi: 10.1016/S1053-4296(03)00073-0, PMID: 14586831

[6] Kapiteijn E Marijnen CA Nagtegaal ID Putter H Steup WH Wiggers T. Preoperative radiotherapy combined with total mesorectal excision for resectable rectal cancer. N Engl J Med. (2001) 345:638–46. doi: 10.1056/NEJMoa010580, PMID: 11547717

[7] Zisis C Tsakiridis K Kougioumtzi I Zarogoulidis P Darwiche K Machairiotis N. The management of the advanced colorectal cancer: management of the pulmonary metastases. J Thorac Dis. (2013) 5:S383–8. doi: 10.3978/j.issn.2072-1439.2013.06.23, PMID: 24102011 PMC3791498

[8] Stillwell AP Ho YH Veitch C. Systematic review of prognostic factors related to overall survival in patients with stage IV colorectal cancer and unresectable metastases. World J Surg. (2011) 35:684–92. doi: 10.1007/s00268-010-0891-8, PMID: 21181473

[9] Mitry E Guiu B Cosconea S Jooste V Faivre J Bouvier AM. Epidemiology, management and prognosis of colorectal cancer with lung metastases: a 30-year population-based study. Gut. (2010) 59:1383–8. doi: 10.1136/gut.2010.211557, PMID: 20732912

[10] Labianca R Beretta GD Kildani B Milesi L Merlin F Mosconi S. Colon cancer. Crit Rev Oncol/Hematol. (2010) 74:106–33. doi: 10.1016/j.critrevonc.2010.01.010, PMID: 20138539

[11] Tampellini M Ottone A Bellini E Alabiso I Baratelli C Bitossi R. The role of lung metastasis resection in improving outcome of colorectal cancer patients: results from a large retrospective study. Oncologist. (2012) 17:1430–8. doi: 10.1634/theoncologist.2012-0142, PMID: 22956535 PMC3500365

[12] Wang Z Wang X Yuan J Zhang X Zhou J Lu M. Survival benefit of palliative local treatments and efficacy of different pharmacotherapies in colorectal cancer with lung metastasis: results from a large retrospective study. Clin Colorectal Cancer. (2018) 17:e233–55. doi: 10.1016/j.clcc.2017.12.005, PMID: 29305209

[13] Riihimäki M Hemminki A Sundquist J Hemminki K. Patterns of metastasis in colon and rectal cancer. Sci Rep. (2016) 6:29765. doi: 10.1038/srep29765, PMID: 27416752 PMC4945942

[14] Imanishi M Yamamoto Y Hamano Y Yamada T Moriwaki T Gosho M. Efficacy of adjuvant chemotherapy after resection of pulmonary metastasis from colorectal cancer: a propensity score–matched analysis. Eur J Cancer. (2019) 106:69–77. doi: 10.1016/j.ejca.2018.10.003, PMID: 30471650

[15] Shin AE Giancotti FG Rustgi AK. Metastatic colorectal cancer: mechanisms and emerging therapeutics. Trends Pharmacol Sci. (2023) 44:222–36. doi: 10.1016/j.tips.2023.01.003, PMID: 36828759 PMC10365888

[16] Biller LH Schrag D. Diagnosis and treatment of metastatic colorectal cancer: A review. JAMA. (2021) 325:669–85. doi: 10.1001/jama.2021.0106, PMID: 33591350

[17] Al Bitar S El-Sabban M Doughan S Abou-Kheir W. Molecular mechanisms targeting drug-resistance and metastasis in colorectal cancer: Updates and beyond. World J Gastroenterol. (2023) 29:1395–426. doi: 10.3748/wjg.v29.i9.1395, PMID: 36998426 PMC10044855

[18] Wang X Zhang J. The treatment strategies for distant metastases of colorectal cancer. Chin J Colorectal Dis. (2016) 04:282–6. doi: 10.3877/cma.j.issn.2095-3224.2016.04.001

[19] Lee S An M Lee J. Immune landscape of colorectal cancer lung metastasis. J Clin Oncol. (2022) . 40:e15542–2. doi: 10.1200/JCO.2022.40.16_suppl.e15542

[20] van Eck NJ Waltman L. Software survey: VOSviewer, a computer program for bibliometric mapping. Scientometrics. (2010) 84:523–38. doi: 10.1007/s11192-009-0146-3, PMID: 20585380 PMC2883932

[21] Thompson DF Walker CK descriptive A. and historical review of bibliometrics with applications to medical sciences. Pharmacotherapy. (2015) 35:551–9. doi: 10.1002/phar.1586, PMID: 25940769

[22] Chen C. Searching for intellectual turning points: progressive knowledge domain visualization. Proc Natl Acad Sci U.S.A. (2004) 101 Suppl 1:5303–10. doi: 10.1073/pnas.0307513100, PMID: 14724295 PMC387312

[23] Chen C. CiteSpace II: Detecting and visualizing emerging trends and transient patterns in scientific literature. J Am Soc Inf Sci Technol. (2006) 57:359–77. doi: 10.1002/asi.20317

[24] Perianes-Rodriguez A Waltman L van Eck NJ. Constructing bibliometric networks: A comparison between full and fractional counting. J Informetr. (2016) 10:1178–95. doi: 10.1016/j.joi.2016.10.006

[25] Qin Y Zhang Q Liu Y. Analysis of knowledge bases and research focuses of cerebral ischemia-reperfusion from the perspective of mapping knowledge domain. Brain Res Bull. (2020) 156:15–24. doi: 10.1016/j.brainresbull.2019.12.004, PMID: 31843561

[26] Arneth B. Tumor microenvironment. Medicina (Kaunas). (2019) 56:15. doi: 10.3390/medicina56010015, PMID: 31906017 PMC7023392

[27] Elia I Haigis MC. Metabolites and the tumor microenvironment: from cellular mechanisms to systemic metabolism. Nat Metab. (2021) 3:21–32. doi: 10.1038/s42255-020-00317-z, PMID: 33398194 PMC8097259

[28] Dongre A Weinberg RA. New insights into the mechanisms of epithelial-mesenchymal transition and implications for cancer. Nat Rev Mol Cell Biol. (2019) 20:69–84. doi: 10.1038/s41580-018-0080-4, PMID: 30459476

[29] Bao Z Zeng W Zhang D Wang L Deng X Lai J. SNAIL induces EMT and lung metastasis of tumours secreting CXCL2 to promote the invasion of M2-type immunosuppressed macrophages in colorectal cancer. Int J Biol Sci. (2022) 18:2867–81. doi: 10.7150/ijbs.66854, PMID: 35541899 PMC9066124

[30] Zhou J. Study on the targeted therapy of colorectal cancer with multiple pathway. 1679 In: BIO Web of Conferences, ICBB 2024, vol. 111. (2024). doi: 10.1051/bioconf/202411102025

[31] Zheng K Zhou X Yu J Li Q Wang H Li M. Epigenetic silencing of miR-490-3p promotes development of an aggressive colorectal cancer phenotype through activation of the Wnt/β-catenin signaling pathway. Cancer Lett. (2016) 376:178–87. doi: 10.1016/j.canlet.2016.03.024, PMID: 27037061

[32] Liaghat M Ferdousmakan S Mortazavi SH Yahyazadeh S Irani A Banihashemi S. The impact of epithelial-mesenchymal transition (EMT) induced by metabolic processes and intracellular signaling pathways on chemo-resistance, metastasis, and recurrence in solid tumors. Cell Commun Signaling. (2024) 22:575. doi: 10.1186/s12964-024-01957-4, PMID: 39623377 PMC11610171

[33] Vincan E Barker N. The upstream components of the Wnt signalling pathway in the dynamic EMT and MET associated with colorectal cancer progression. Clin Exp Metastasis. (2008) 25:657–63. doi: 10.1007/s10585-008-9156-4, PMID: 18350253

[34] Lu T Zheng C Fan Z. Cardamonin suppressed the migration, invasion, epithelial mesenchymal transition (EMT) and lung metastasis of colorectal cancer cells by down-regulating ADRB2 expression. Pharm Biol. (2022) 60:1011–21. doi: 10.1080/13880209.2022.2069823, PMID: 35645356 PMC9154753

[35] Shu J Wang L Han F Chen Y Wang S Luo F. BTBD7 downregulates E-cadherin and promotes epithelial-mesenchymal transition in lung cancer. BioMed Res Int. (2019) 2019:1–11. doi: 10.1155/2019/5937635, PMID: 31886230 PMC6900955

[36] Herszényi L Hritz I Lakatos G Varga MZ Tulassay Z. The behavior of matrix metalloproteinases and their inhibitors in colorectal cancer. Int J Mol Sci. (2012) 13:13240–63. doi: 10.3390/ijms131013240, PMID: 23202950 PMC3497324

[37] Oshima T Kunisaki C Yoshihara K Yamada R Yamamoto N Sato T. Clinicopathological significance of the gene expression of matrix metalloproteinases and reversion-inducing cysteine-rich protein with Kazal motifs in patients with colorectal cancer: MMP-2 gene expression is a useful predictor of liver metastasis from colorectal cancer. Oncol Rep. (2008) 19:1285–91. doi: 10.3892/or.19.5.1285, PMID: 18425389

[38] Kostova E Slaninka-Miceska M Labacevski N Jakovski K Trojachanec J Atanasovska E. Expression of matrix metalloproteinases 2, 7 and 9 in patients with colorectal cancer. Vojnosanit Pregl. (2014) 71:52–9. doi: 10.2298/vsp121221024k, PMID: 24516991

[39] Crosas-Molist E Samain R Kohlhammer L Orgaz JL George SL Maiques O. Rho GTPase signaling in cancer progression and dissemination. Physiol Rev. (2022) 102:455–510. doi: 10.1152/physrev.00045.2020, PMID: 34541899

[40] Sahai E Marshall CJ. RHO-GTPases and cancer. Nat Rev Cancer. (2002) 2:133–42. doi: 10.1038/nrc725, PMID: 12635176

[41] Bedi A Pasricha P Akhtar A Barber J Bedi GC Giardiello F. Inhibition of apoptosis during development of colorectal cancer. Cancer Res. (1995) 55:1811–6. 7728743

[42] Mortenson MM Schlieman MG Virudalchalam S Bold RJ. Overexpression of BCL-2 results in activation of the AKT/NF-kB Cell survival pathway. J Surg Res. (2003) 114:302. doi: 10.1016/j.jss.2003.08.103

[43] Osaki M Kase S Adachi K Takeda A Hashimoto K Ito H. Inhibition of the PI3K-Akt signaling pathway enhances the sensitivity of Fas-mediated apoptosis in human gastric carcinoma cell line, MKN-45. J Cancer Res Clin Oncol. (2004) 130:8–14. doi: 10.1007/s00432-003-0505-z, PMID: 14605879 PMC12161786

[44] Chen S Gong Y Shen Y Liu Y Fu Y Dai Y. INHBA is a novel mediator regulating cellular senescence and immune evasion in colorectal cancer. J Cancer. (2021) 12:5938–49. doi: 10.7150/jca.61556, PMID: 34476008 PMC8408109

[45] Zhou J Zhang M Huang Y Feng L Chen H Hu Y. MicroRNA-320b promotes colorectal cancer proliferation and invasion by competing with its homologous microRNA-320a. Cancer Lett. (2015) 356:669–75. doi: 10.1016/j.canlet.2014.10.014, PMID: 25458952 PMC4397650

[46] Liang Z Brooks J Willard M Liang K Yoon Y Kang S. CXCR4/CXCL12 axis promotes VEGF-mediated tumor angiogenesis through Akt signaling pathway. Biochem Biophys Res Commun. (2007) 359:716–22. doi: 10.1016/j.bbrc.2007.05.182, PMID: 17559806 PMC1986788

[47] Khare T Bissonnette M Khare S. CXCL12-CXCR4/CXCR7 axis in colorectal cancer: therapeutic target in preclinical and clinical studies. Int J Mol Sci. (2021) 22:7371. doi: 10.3390/ijms22147371, PMID: 34298991 PMC8305488

[48] Cao G Cheng D Ye L Pan Y Yang F Lyu S. Surgical resection of pulmonary metastases from colorectal cancer: 11 years of experiences. PloS One. (2017) 12:e0175284. doi: 10.1371/journal.pone.0175284, PMID: 28394911 PMC5386242

[49] Baron O Amini M Duveau D Despins P Sagan CA Michaud JL. Surgical resection of pulmonary metastases from colorectal carcinoma. Five-year survival and main prognostic factors. Eur J Cardiothorac Surg. (1996) 10:347–51. doi: 10.1016/s1010-7940(96)80093-5, PMID: 8737691

[50] Iida T Nomori H Shiba M Nakajima J Okumura S Horio H. Prognostic factors after pulmonary metastasectomy for colorectal cancer and rationale for determining surgical indications: a retrospective analysis. Ann Surg. (2013) 257:1059–64. doi: 10.1097/SLA.0b013e31826eda3b, PMID: 23001087

[51] Watanabe K Nagai K Kobayashi A Sugito M Saito N. Factors influencing survival after complete resection of pulmonary metastases from colorectal cancer. Br J Surg. (2009) 96:1058–65. doi: 10.1002/bjs.6682, PMID: 19672932

[52] Schüle S Dittmar Y Knösel T Krieg P Albrecht R Settmacher U. Long-term results and prognostic factors after resection of hepatic and pulmonary metastases of colorectal cancer. Int J Colorectal Dis. (2013) 28:537–45. doi: 10.1007/s00384-012-1553-0, PMID: 22885838

[53] Pfannschmidt J Dienemann H Hoffmann H. Surgical resection of pulmonary metastases from colorectal cancer: A systematic review of published series. Ann Thorac Surg. (2007) 84:324–38. doi: 10.1016/j.athoracsur.2007.02.093, PMID: 17588454

[54] Milosevic M Edwards J Tsang D Dunning J Shackcloth M Batchelor T. Pulmonary Metastasectomy in Colorectal Cancer: updated analysis of 93 randomized patients – control survival is much better than previously assumed. Colorectal Dis. (2020) 22:1314. doi: 10.1111/codi.15113, PMID: 32388895 PMC7611567

[55] King J Glenn D Clark W Zhao J Steinke K Clingan P. Percutaneous radiofrequency ablation of pulmonary metastases in patients with colorectal cancer. Br J Surg. (2004) 91:217–23. doi: 10.1002/bjs.4392, PMID: 14760671

[56] Tomimaru Y Yasumoto T Matsunaga H Ide Y Ikeda N Maruyama K. Two cases of pulmonary metastases from colorectal cancer successfully treated by radiofrequency ablation therapy. Gan To Kagaku Ryoho. (2007) 34:2029–31., PMID: 18219888

[57] Yamakado K Hase S Matsuoka T Tanigawa N Nakatsuka A Takaki H. Radiofrequency ablation for the treatment of unresectable lung metastases in patients with colorectal cancer: a multicenter study in Japan. J Vasc Interv Radiol. (2007) 18:393–8. doi: 10.1016/j.jvir.2006.11.003, PMID: 17377185

[58] Yan TD King J Sjarif A Glenn D Steinke K Morris DL. Percutaneous radiofrequency ablation of pulmonary metastases from colorectal carcinoma: prognostic determinants for survival. Ann Surg Oncol. (2006) 13:1529–37. doi: 10.1245/s10434-006-9101-1, PMID: 17009153

[59] Lencioni R Crocetti L Cioni R Suh R Glenn D Regge D. Response to radiofrequency ablation of pulmonary tumours: a prospective, intention-to-treat, multicentre clinical trial (the RAPTURE study). Lancet Oncol. (2008) 9:621–8. doi: 10.1016/S1470-2045(08)70155-4, PMID: 18565793

[60] Ishikawa K Hashiguchi Y Mochizuki H Ozeki Y Ueno H. Extranodal cancer deposit at the primary tumor site and the number of pulmonary lesions are useful prognostic factors after surgery for colorectal lung metastases. Dis Colon Rectum. (2003) 46:629–36. doi: 10.1007/s10350-004-6623-0, PMID: 12792439

[61] Girard P Ducreux M Baldeyrou P Rougier P Le Chevalier T Bougaran J. Surgery for lung metastases from colorectal cancer: analysis of prognostic factors. J Clin Oncol. (1996) 14:2047–53. doi: 10.1200/JCO.1996.14.7.2047, PMID: 8683235

[62] Yang XQ Chen C Hou JX Peng CW Huang CQ Li Y. Preoperative serum carbohydrate antigen 242 is a useful predictive and prognostic marker in colorectal cancer. Hepatogastroenterology. (2011) 58:377–82., PMID: 21661399

[63] Chen J Zhao G. An urgent need to develop novel markers for prognosis in colon cancer. J Surg Oncol. (2010) 102:295–6. doi: 10.1002/jso.21615, PMID: 20740590

[64] Messersmith WA Jimeno A Jacene H Zhao M Kulesza P Laheru DA. Phase I trial of oxaliplatin, infusional 5-fluorouracil, and leucovorin (FOLFOX4) with erlotinib and bevacizumab in colorectal cancer. Clin Colorectal Cancer. (2010) 9:297–304. doi: 10.3816/CCC.2010.n.043, PMID: 21208844 PMC3033228

[65] Hurwitz H Fehrenbacher L Novotny W Cartwright T Hainsworth J Heim W. Bevacizumab plus irinotecan, fluorouracil, and leucovorin for metastatic colorectal cancer. N Engl J Med. (2004) 350:2335–42. doi: 10.1056/NEJMoa032691, PMID: 15175435

[66] Cui F Chen Jz Wan C Chen B Luo R Zheng H. Clinical research of bevacizumab in combination with irinotecan, fluorouracil and leucovorin for advanced metastatic colorectal cancer. Zhonghua Wei Chang Wai Ke Za Zhi. (2009) 12:374–7. doi: 10.3760/CMA.J.ISSN.1671-0274.2009.04.015, PMID: 19598023

[67] Yasuda S Kamata H Machida T Okada K Tanaka A Suzuki T. A case of isolated paraaortic lymph node recurrence from colon cancer successfully treated with chemoradiotherapy. Tokai J Exp Clin Med. (2012) 37:47–50., PMID: 22763827

[68] Li W Peng J Li C Yuan L Fan Wh Pan Z. Prognosis and risk factors for the development of pulmonary metastases after preoperative chemoradiotherapy and radical resection in patients with locally advanced rectal cancer. Ann Transl Med. (2020) 8:117. doi: 10.21037/atm.2019.12.108, PMID: 32175410 PMC7049015

[69] Hohla F Mayer P Hutter J Meißnitzer T Greilet R. A 55-year-old woman with locally advanced rectal cancer and a resectable synchronous hepatic metastasis: a case report. Memo. (2012) 5:273–6. doi: 10.1007/s12254-012-0060-x

[70] Russo V Lallo E Munnia A Spedicato M Messerini L D’Aurizio R. Artificial intelligence predictive models of response to cytotoxic chemotherapy alone or combined to targeted therapy for metastatic colorectal cancer patients: A systematic review and meta-analysis. Cancers (Basel). (2022) 14:4012. doi: 10.3390/cancers14164012, PMID: 36011003 PMC9406544

[71] Taylor JC Swinson D Seligmann JF Birch RJ Dewdney A Brown V. Addressing the variation in adjuvant chemotherapy treatment for colorectal cancer: Can a regional intervention promote national change? Int J Cancer. (2021) 148:845–56. doi: 10.1002/ijc.33261, PMID: 32818319

[72] Guerrera F Mossetti C Ceccarelli M Bruna MC Bora G Olivetti S. Surgery of colorectal cancer lung metastases: analysis of survival, recurrence and re-surgery. J Thorac Dis. (2016) 8:1764. doi: 10.21037/jtd.2016.05.98, PMID: 27499967 PMC4958892

[73] Rajakannu M Magdeleinat P Vibert E Ciacio O Pittau G Innominato P. Is cure possible after sequential resection of hepatic and pulmonary metastases from colorectal cancer? Clin Colorectal Cancer. (2018) 17:41–9. doi: 10.1016/j.clcc.2017.06.006, PMID: 28709876

[74] Shibutani M Maeda K Nagahara H Fukuoka T Iseki Y Matsutani S. Complete response of pulmonary metastases from rectal cancer to tegafur-uracil/leucovorin plus bevacizumab in an elderly patient: A case report. Case Rep Oncol. (2018) 11:461–6. doi: 10.1159/000490698, PMID: 30057541 PMC6062657

[75] Shiomi K Naito M Sato T Nakamura T Nakashima H Naito M. Effect of adjuvant chemotherapy after pulmonary metastasectomy on the prognosis of colorectal cancer. Ann Med Surg (Lond). (2017) 20:19–25. doi: 10.1016/j.amsu.2017.06.026, PMID: 28702182 PMC5484968

[76] Martínez-Pérez J Torrado C Domínguez-Cejudo MA Valladares-Ayerbes M. Targeted treatment against cancer stem cells in colorectal cancer. Int J Mol Sci. (2024) 25:6220. doi: 10.3390/ijms25116220, PMID: 38892410 PMC11172446

[77] Tunney R Gnananandha D Shah R Taggart S. P195 Temporal trends and distribution of recurrent disease following lung cancer surgery and relationship to pre-operative PET scan. Thorax. (2010) 65:159–60. doi: 10.1136/thx.2010.151043.46

[78] Baron O Hamy A Roussel JC Galetta D al Habash O Duveau D. Surgical treatment of pulmonary metastases of colorectal cancers. 8-year survival and main prognostic factors. Rev Mal Respir. (1999) 16:809–15., PMID: 10612150

[79] Ohlsson L Lindmark G Israelsson A Palmqvist R Öberg Å Hammarström ML. Lymph node tissue kallikrein-related peptidase 6 mRNA: a progression marker for colorectal cancer. Br J Cancer. (2012) 107:150–7. doi: 10.1038/bjc.2012.220, PMID: 22699826 PMC3389417

[80] Yu WS Bae MK Choi JK Hong YK Park IK. Pulmonary metastasectomy in colorectal cancer: A population-based retrospective cohort study using the korean national health insurance database. Cancer Res Treat. (2021) 53:1104–12. doi: 10.4143/crt.2020.1213, PMID: 33494126 PMC8524016

[81] Shalabi A Ehab A Shalabi SF Kugler G Schäfers HJ Graeter T. Laser assisted pulmonary metastasectomy promises a low local recurrence rate. Sci Rep. (2024) 14:5988. doi: 10.1038/s41598-024-56566-5, PMID: 38472291 PMC10933436

[82] Cucchetti A Piscaglia F Cescon M Colecchia A Ercolani G Bolondi L. Cost-effectiveness of hepatic resection versus percutaneous radiofrequency ablation for early hepatocellular carcinoma. J Hepatol. (2013) 59:300–7. doi: 10.1016/j.jhep.2013.04.009, PMID: 23603669

[83] Cho YK Kim JK Kim WT Chung JW. Hepatic resection versus radiofrequency ablation for very early stage hepatocellular carcinoma: a Markov model analysis. Hepatology. (2010) 51:1284–90. doi: 10.1002/hep.23466, PMID: 20099299

[84] Yang Z Lyu X Yang H Wang B Xu D Huo L. Survival after radiofrequency ablation and/or chemotherapy for lung cancer and pulmonary metastases: a systematic review and meta-analysis. Front Immunol. (2023) 14:1240149. doi: 10.3389/fimmu.2023.1240149, PMID: 37869011 PMC10587578

[85] Fenton HM Finan PJ Milton R Shackcloth M Taylor JC Treasure T. National variation in pulmonary metastasectomy for colorectal cancer. Colorectal Dis. (2021) 23:1306–16. doi: 10.1111/codi.15506, PMID: 33368958 PMC8614123

[86] Treasure T Farewell V Macbeth F Batchelor T Milošević M King J. The Pulmonary Metastasectomy in Colorectal Cancer cohort study: Analysis of case selection, risk factors and survival in a prospective observational study of 512 patients. Colorectal Dis. (2021) 23:1793–803. doi: 10.1111/codi.15651, PMID: 33783109 PMC8496511

[87] Zurlo IV Calegari MA Congedo MT Basso M Vita ML Petracca Ciavarella L. A retrospective analysis of real-life management of colorectal cancer lung-limited metastases treated with surgery: outcomes and prognostic factors. J Clin Med. (2024) 13:6651. doi: 10.3390/jcm13226651, PMID: 39597795 PMC11594730

[88] Ziranu P Ferrari PA Guerrera F Bertoglio P Tamburrini A Pretta A. Clinical score for colorectal cancer patients with lung-limited metastases undergoing surgical resection: Meta-Lung Score. Lung Cancer. (2023) 184:107342. doi: 10.1016/j.lungcan.2023.107342, PMID: 37573705

[89] Wang R Dai W Gong J Huang M Hu T Li H. Development of a novel combined nomogram model integrating deep learning-pathomics, radiomics and immunoscore to predict postoperative outcome of colorectal cancer lung metastasis patients. J Hematol Oncol. (2022) 15:11. doi: 10.1186/s13045-022-01225-3, PMID: 35073937 PMC8785554

