# Supplementary figures and images for: Decoding colorectal cancer lung metastasis: a global research odyssey
Source: Front Oncol. 2025 Jul 24;15:1587422. doi: 10.3389/fonc.2025.1587422 (PMC12328170; doi:10.3389/fonc.2025.1587422)

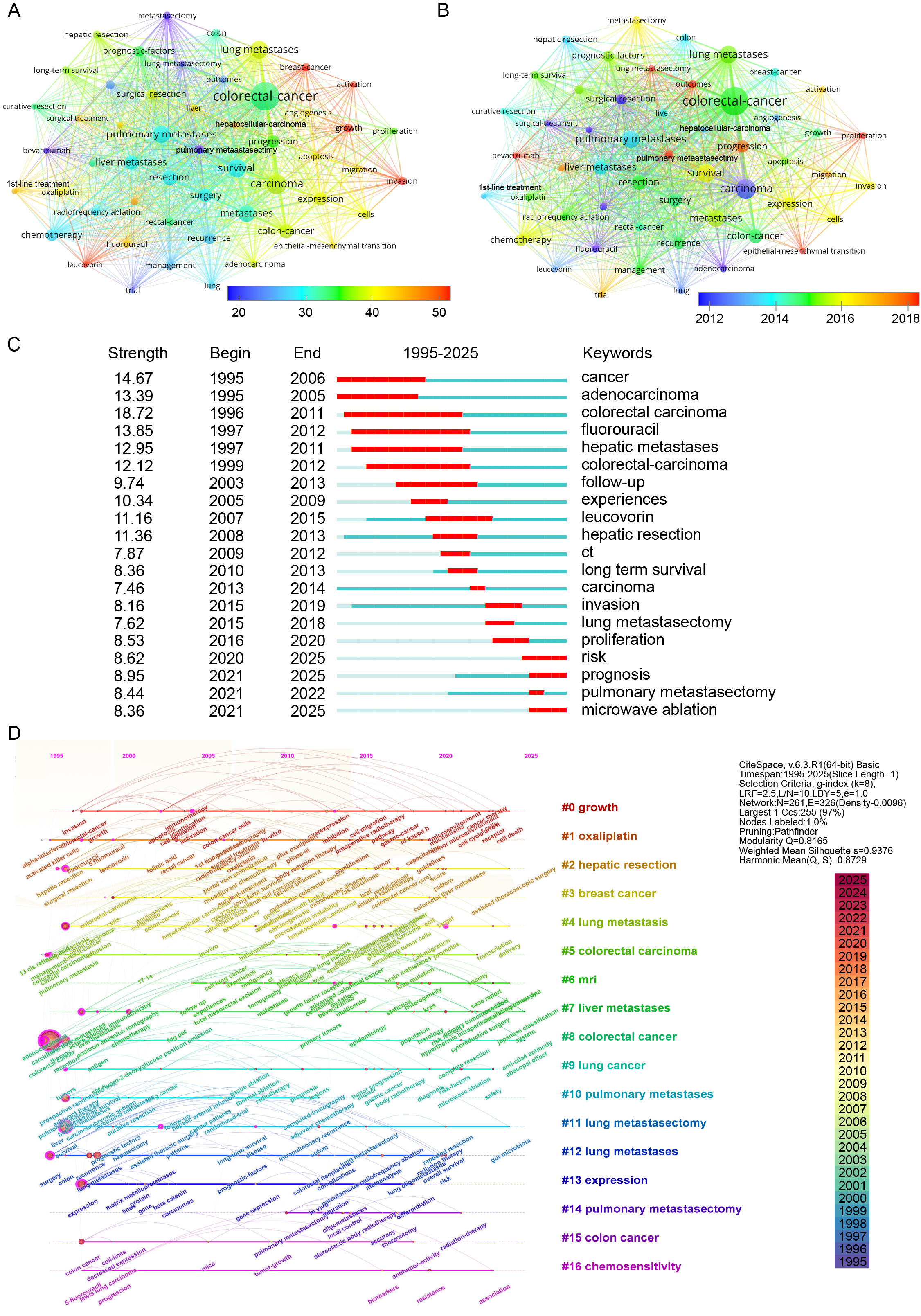

Supplement: Supplementary Figure 1 — Visualization analysis of keyword citation impact, research hotspot evolution, and temporal trends in studies on colorectal cancer lung metastases. (A) Overlay visualization of the average citation counts for the selected keywords. (B) Overlay visualization depicting the average publication year of the keywords. (C) Top 20 keywords exhibiting the highest citation bursts. (D) Timeline and keyword clustering diagram illustrating research trends in colorectal cancer lung metastases. [file Image1.tif]

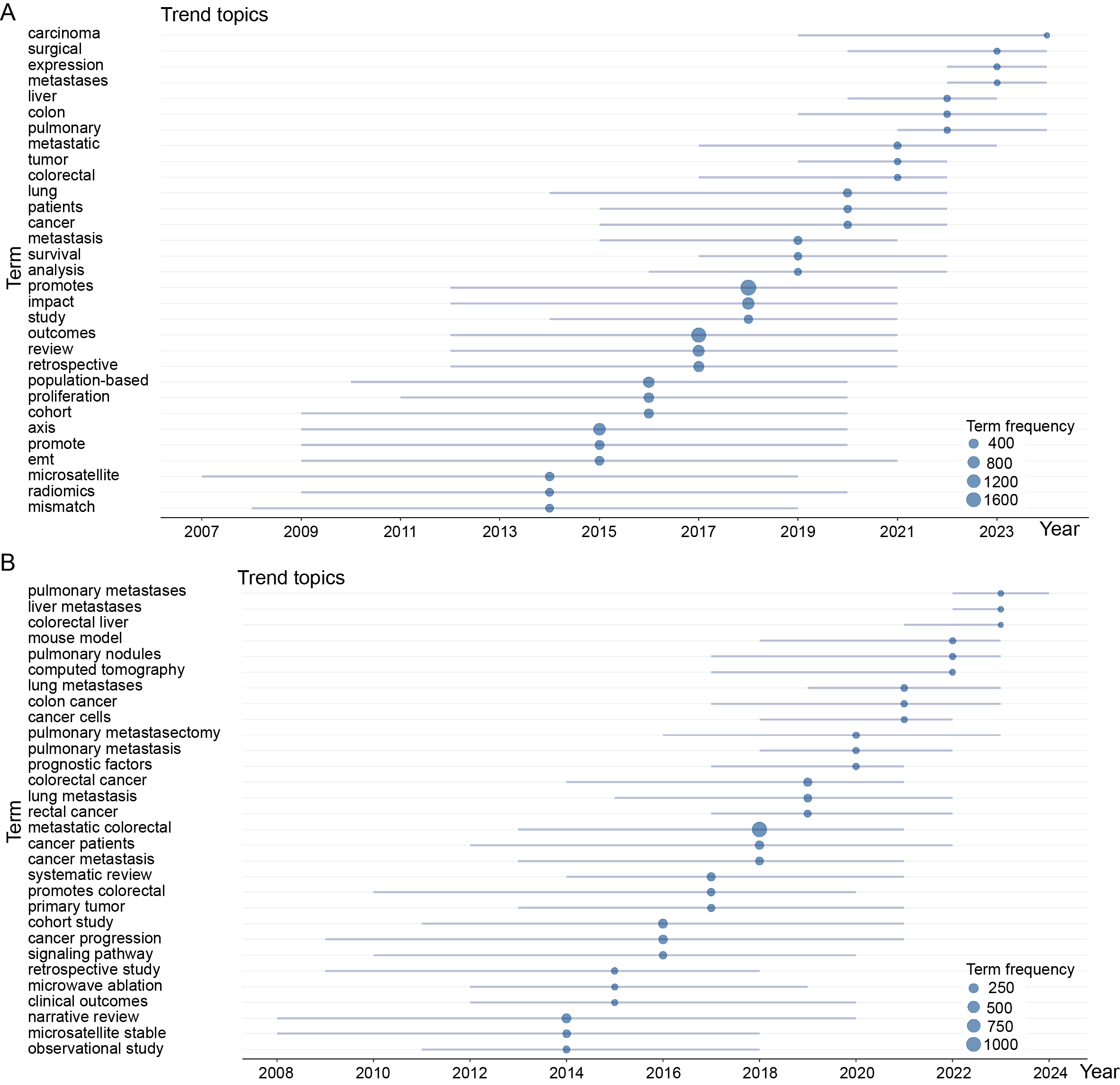

Supplement: Supplementary Figure 2 — Thematic trends and their temporal evolution in colorectal cancer lung metastasis research. (A) Emerging research trends in the field. (B) Advancements in research within the field and related domains. [file Image2.tif]
